# Dr. George W. Sayles

**Published:** 1911-09

**Authors:** 


Dr. George W. Sayles died on August 6 at his home, 211 Amherst Street, Buffalo, after an illness of several months.

Dr. Sayles was born in Branchton, Ont., and received his early education in his home town. He later studied pharmacy at Toronto. He came to Buffalo 32 years ago and opened a drug store at Amherst street and Military road. In 1890 he entered the medical department of the University of Buffalo being graduated in 1893.

The same year, while an interne at the General Hospital, he married Miss Etta T. Twaits, a nurse on the hospital staff.

Besides Mrs. Sayles and his daughter, Vera M. Sayles, Dr. Sayles is survived by his father, Peter F. Sayles and two brothers, James G. and William C. Sayles, all of this city. The funeral was held from the family home. Burial was at Forest Lawn.

